# Scalable Textile Manufacturing Methods for Fabricating Triboelectric Nanogenerators with Balanced Electrical and Wearable Properties

**DOI:** 10.1021/acsaelm.1c01095

**Published:** 2022-01-26

**Authors:** K. R. Sanjaya Gunawardhana, Nandula D. Wanasekara, Kahagala Gamage Wijayantha, R. D. Ishara Dharmasena

**Affiliations:** † Department of Textile and Apparel Engineering, Faculty of Engineering, University of Moratuwa, Bandaranayake Mawatha, Moratuwa 10400, Sri Lanka; ‡ Energy Research Laboratory, Department of Chemistry, Loughborough University, Loughborough, Leicestershire LE11 3TU, United Kingdom; § Wolfson School of Mechanical Electrical and Manufacturing Engineering, Loughborough University, Loughborough, Leicestershire LE11 3TU, United Kingdom

**Keywords:** energy harvesting, triboelectric nanogenerators, smart textiles, textile TENG, DDEF model, scalable TENG

## Abstract

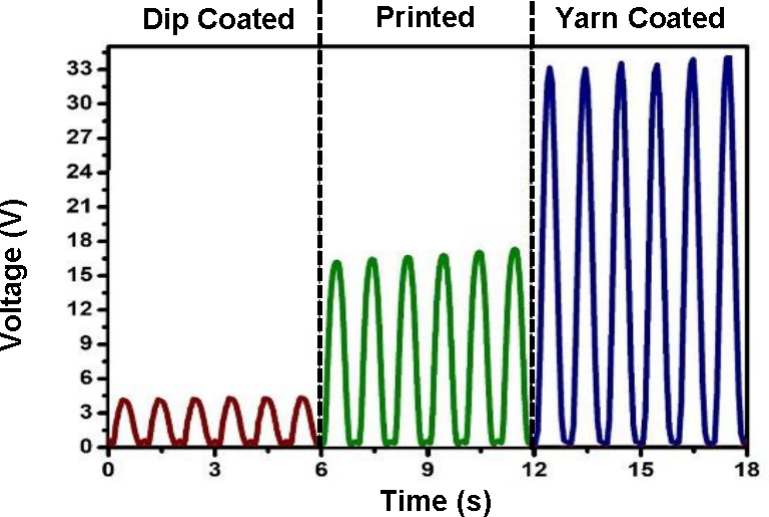

Triboelectric nanogenerators (TENGs) are foreseen as a leading candidate to harvest mechanical energy from ambient sources such as human body movements. However, wearable TENGs, which are used for this purpose, require adequate wearability for long durations, in addition to sufficient electrical outputs. So far, it has been difficult to achieve this through the predominantly plastic-based wearable TENGs constructed using conventional nanogenerator fabrication methods. This Article evaluates the use of textile materials and scalable fabrication techniques to develop TENGs targeting balanced electrical and wearable properties. The fabrication process is conducted using yarn-coating, dip-coating, and screen-printing techniques, which are common textile manufacturing methods, and converted into fabrics using flat-bed knitting, resulting in TENGs with improved wearable and electrical performances. The electrical properties (open circuit voltage (*V*_oc_), short circuit current (*I*_sc_), and short circuit charge (*Q*_sc_)) and wearable properties (air permeability, stretch and recovery, and moisture management) of these structures are evaluated, during which the yarn-coated TENG resulted in maximum electrical outputs recording *V*_oc_ ≈ 35 V, *I*_sc_ ≈ 60 nA, and *Q*_sc_ ≈ 12 nC, under mild excitations. In terms of wearability, the yarn-coated TENG again performed exceptionally during the majority of tests providing the best moisture management, air permeability (101 cm^3^/cm^2^/s), and stretch (∼75%), thus proving its suitability for wearable TENG applications.

## Introduction

Advances on the Internet of things (IoT), 5G technology, and artificial intelligence (AI) are shaping to improve the quality of life of the global population.^1−5^ Being connected to the human body, wearable electronics play a key part in facilitating these enhancements^1^ through applications in communication, security, healthcare, personal electronics, and sports.^3,5−8^ However, most wearable electronics are powered with batteries that present problems associated with low flexibility, high rigidity, lack of autonomy and biocompatibility, and increased weight. This not only disturbs the maintenance-free operation of the electronics, but also impairs their wearability.^6^ An ideal solution would be to integrate electronics with energy harvesting systems that capture free energy from the surroundings and convert into electricity in real-time, or to create self-powered electronics that power their own operations, without compromising wearable performances.

Human movement, when converted into electricity, is a ubiquitous energy source for wearable electronics.^9,10^ Triboelectric nanogenerators (TENGs) have emerged as a promising mechanical energy harvesting method for such wearable applications.^1,11,12^ Some of the TENG designs have recorded significant peak power outputs and instantaneous energy conversion efficiencies especially at low frequency movements such as human motion.^1,13,14^ Furthermore, TENGs can be constructed at low cost using widely available and biocompatible materials that are of light weight and have flexibility.^15−17^ These features make TENG a desirable candidate to harvest energy from human movements for wearables, as compared to other methods such as piezoelectric and electromagnetic technologies.^18−21^ More recently, there is increasing interest in developing hybrid energy harvesting systems, where TENG, piezoelectric, and electromagnetic techniques are integrated into the same device structure to improve the overall energy harvesting efficiency.^15,22−24^

Nevertheless, TENG technology for wearable applications (i.e., wearable TENGs) is still at its infancy with several drawbacks.^1,25^ The majority of wearable TENGs have been constructed using flexible plastic sheets, with planar device architectures. These devices do not fit conformally with the 3D shaped human body and lack the air permeability, moisture absorption, washability, and comfort that are essential for prolonged wearability.^26,27^

However, many textile materials that are known to have excellent wearable and comfort characteristics are listed in the triboelectric series.^28,29^ By using textile materials (in fiber, yarn, and fabric forms) as triboelectric active surfaces in TENGs (i.e., textile TENGs), their inherent triboelectric properties can be exploited for electricity generation.^1,30^ Concurrently, if the TENG fabrication process is designed to preserve the wearable properties of these textiles, the resultant devices will overcome many of the aforementioned drawbacks of the wearable TENG technology. Despite recent reports of textile TENGs reaching progressively increasing electrical outputs, there has been limited emphasis on achieving a balance between their electrical and wearable characteristics simultaneously.^1^ Therefore, there is an urgent need to develop textile TENG device designs and fabrication methods, which can provide acceptable levels of electrical and wearable performances for TENGs to become a suitable technology for everyday use.

Furthermore, scaling up of such textile-based wearable TENG designs is also a significant challenge. At present, even the primitive textile TENGs are created via limited lab scale techniques.^1^ Textile manufacturing is a vast industry with well-established production and engineering techniques for textile materials; therefore, it is pivotal to adapt these techniques for textile TENG development to ensure their scalability, potential mass-scale manufacture, and commercialization.^31^

**Figure 1:**
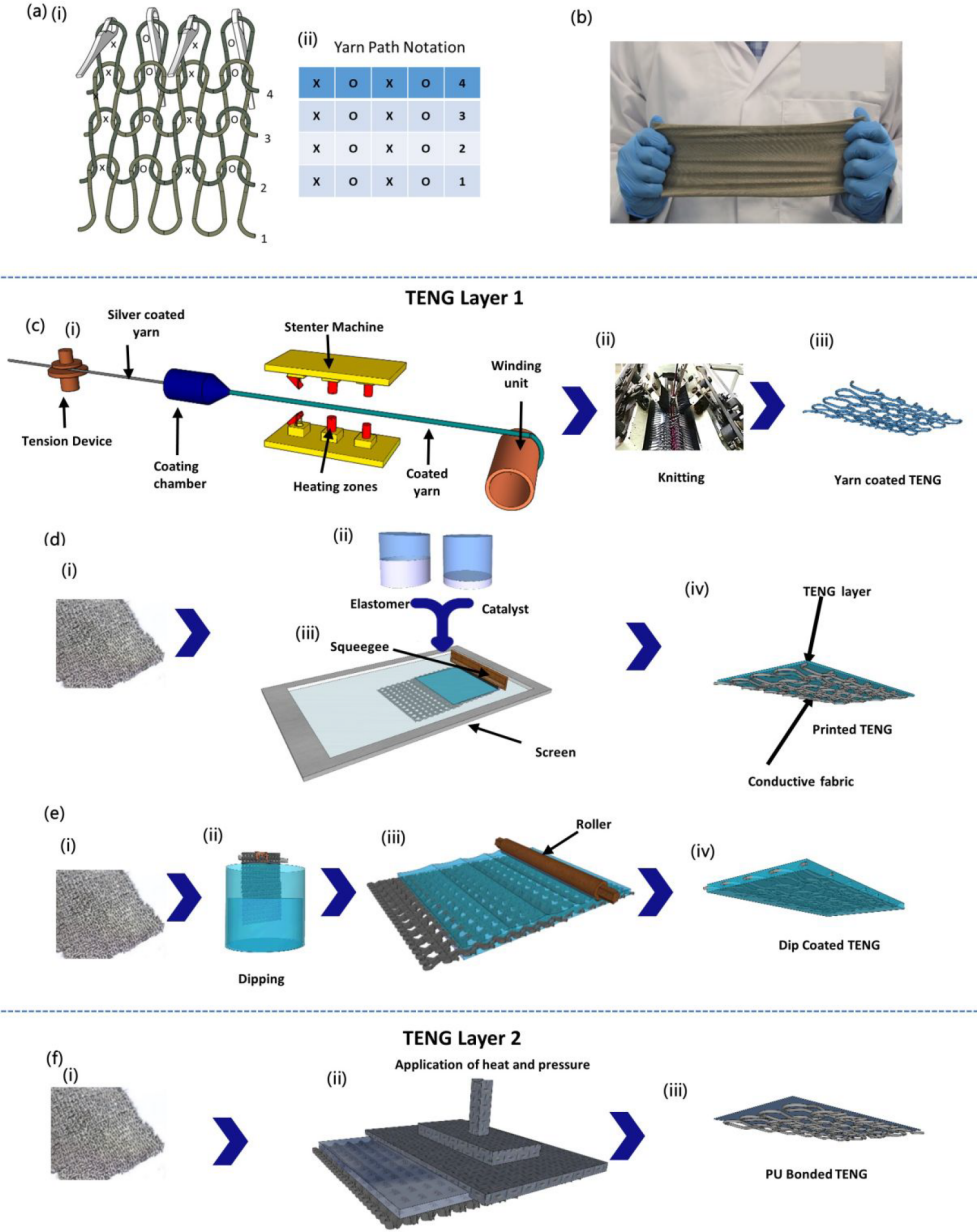
Steps of the TENG fabrication process. (a) The rib knit structure used for TENG fabrics showing (i) a schematic of the construction, and (ii) yarn path notation used to construct the fabric via flat knitting machine. (b) Photograph of a stretchable knitted rib fabric. (c–e) TENG layer 1 preparation: (c) yarn-coating process showing (i) a schematic of the coating setup, (ii) the flatbed knitting machine used for fabric construction, and (iii) a schematic of the yarn-coated TENG. (d) Screen-printing process showing (i) the conductive fabric, (ii) PDMS elastomer and catalyst mixing, (iii) a schematic of the screen-printing setup, and (iv) the screen-printed TENG. (e) Dip-coating setup depicting (i) the conductive fabric, (ii) the fabric dipping process, (iii) removal of excessive PDMS via rolling, and (iv) the dip-coated TENG. (f) TENG layer 2 preparation: polyurethane (PU) heat bonding process showing (i) the conductive fabric, (ii) application of heat and pressure for bonding, and (iii) the PU-bonded TENG.

Addressing these three key challenges, in this work, we evaluate the use of common textile materials and commercial textile manufacturing techniques to fabricate a TENG with balanced electrical and wearable properties. A series of commercial textile processing techniques including yarn coating, dip coating, and screen printing are used to enhance the triboelectric characteristics of the textiles. The triboelectric fabrics, which function as the active elements of the final TENG design, are constructed using flatbed knitting (with a rib knit structure) (Figure 1a,b), targeting improved electrical performance.^32−34^ The textile TENGs are examined for their electrical properties using previously established current, charge, and voltage output characterization techniques.^2,35−37^ Finally, the wearable properties of the textile TENGs are assessed in a textile testing laboratory using standard test methods.

This work, therefore, evaluates the textile TENG fabrication from the yarn stage to final textile products, paving the path toward their scaling up and potential practical applications.

## Experimental Methods

### Conductive Substrate Yarn

A silver-plated nylon continuous filament yarn (875 dTex, Kitronik Ltd. (UK)) was used as the substrate material and the electrode for the TENGs. The selection of a continuous filament yarn (instead of a staple spun yarn) was to provide improved conductivity and continuity of the TENG output signals.^38^

### Triboelectric Layer 1

Polydimethylsiloxane (PDMS) (Sylgard 186; Dow Corning (U.S.)), which is commonly used for smart textiles, was used as the coating material for the first triboelectric layer. PDMS elastomer (base) and cross-linker (curing agent) were mixed (10:1 weight ratio) and kept (1 h) inside a vacuum oven to remove the trapped air. This PDMS mixture was applied on the textile (yarn/fabric) using three different processing techniques, yarn coating,^39^ screen printing,^13^ and dip coating,^40^ as described below.

#### Method (i): Yarn Coating

A scalable method similar to industrial yarn coating, which provides batch processability and knittability, was used for the yarn coating in this work (Figure 1c). Once coated with PDMS, the yarn was passed through a heating chamber (miniaturized industrial stenter) at 150 °C for 6 min, for curing. We note that such heating chambers are readily available in large scale in the textile wet processing industry and can be customized for different material types and treatment processes.^41^ Furthermore, the use of Sylgard 186 PDMS material made the yarn treatment and the curing process more convenient due to the relatively high viscosity, and, together with the design of the yarn coating and heat chamber setup, helped to counter the adverse effects of gravity during the yarn-coating process.

The coated yarn was knitted into a fabric (8 cm × 8 cm) using a commercial flat-knitting machine (gauge (needles per inch) = 5) (Figure 1c,ii). A rib knit structure (1 × 1) was selected for all TENG fabric samples developed in this work, targeting high electrical outputs that rib knits are known to provide due to properties such as stretchability^33^ (Figure 1a,b).

#### Method (ii): Screen Printing

A similar fabric structure (1 × 1 rib, 8 cm × 8 cm, gauge 5 knitting) was developed using the conductive yarn, and this fabric was used as the substrate for the screen-printing of PDMS (Figure 1d,i). A 64 count (holes per square inch) commercial screen-printing mesh was used to apply a uniform coating of PDMS on the top surface of the fabric using a screen print setup (Figure 1d,iii), with 5 N force and 7 rpm printing speed. The printed fabric was cured at 150 °C for 6 min.

#### Method (iii): Dip Coating

A fabric with specifications similar to those of method (ii) above was constructed (Figure 1e,i) using the conductive yarns and immersed inside a PDMS bath to simulate industrial fabric dip coating. Following the dipping step (Figure 1e,ii), the excess PDMS was removed through a controlled rolling process (Figure 1e,iii) to obtain a PDMS uptake comparable to that of (i) and (ii). The dip-coated fabric was cured at 150 °C for 6 min.

It should be noted that the curing temperature (150 °C) and the time (6 min) for PDMS used in this study were selected to be well within the specified curing conditions (150 °C for 15 min) by the manufacturer, to ensure that the PDMS coating would contain the required flexibility after curing.

TENG layers produced in methods (i), (ii), and (iii) were tested against identical counter TENG surfaces (triboelectric layer 2) using a vertical contact-separation TENG (VCSTENG) architecture to evaluate the TENG performances.

### Triboelectric Layer 2

The conductive yarn was used to construct a knitted fabric (similar to the substrate of (ii) and (iii) above) as the fabric substrate for triboelectric layer 2 (Figure 1f,i). This fabric was combined with a polyurethane (PU) layer on the top surface. Herein, following a common industrial method, a Bemis TP315 PU bonding layer (thickness ∼100 μm) was placed on the fabric substrate and subjected to 150 °C at 40 kPa for 120–150 s to facilitate heat bonding between the PU and the fabric (Figure 1f,ii).

### Surface Analysis

**Figure 2:**
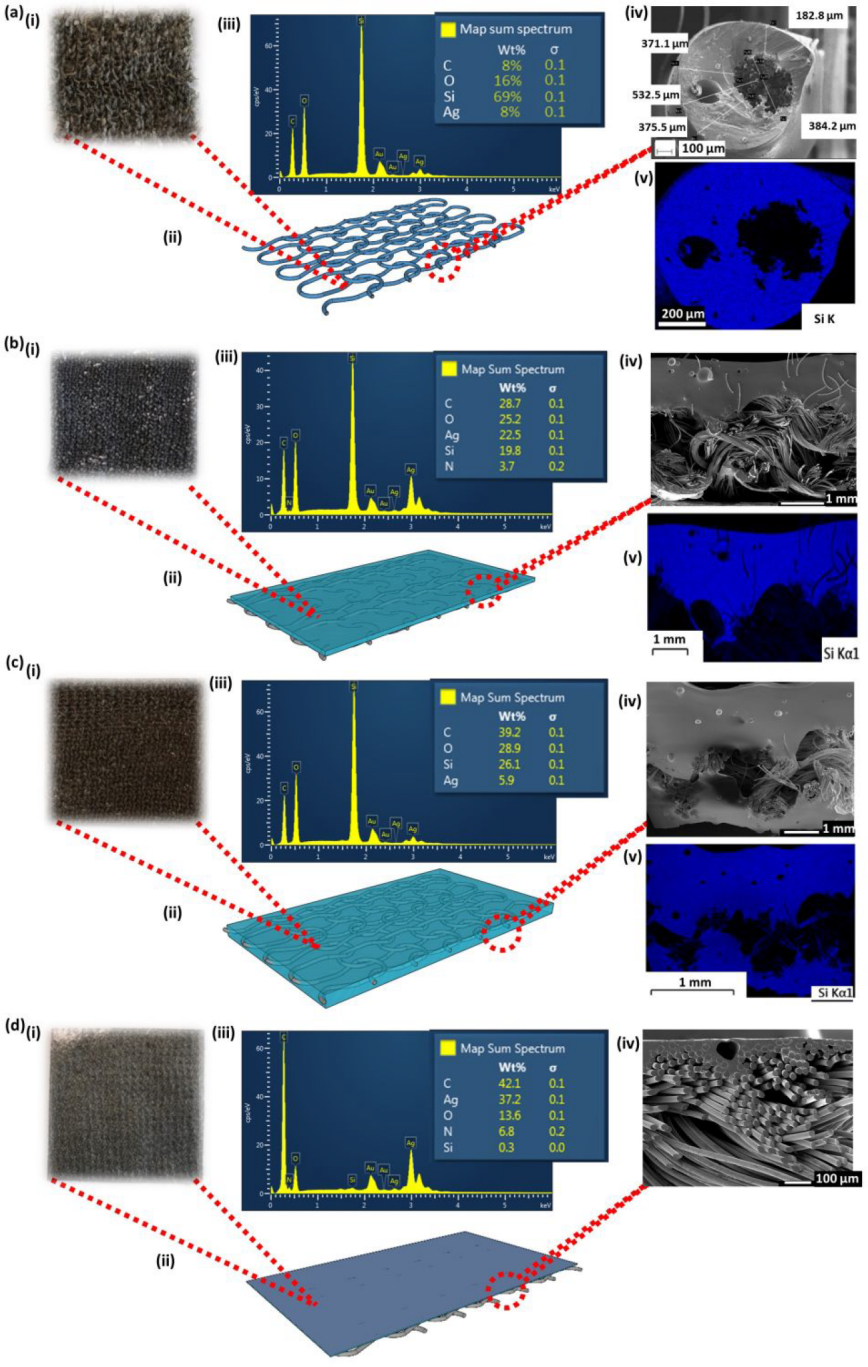
Surface analysis of the TENGs. (a) Yarn-coated TENG layer shown through (i) a photograph, (ii) a schematic, (iii) EDX surface analysis, (iv) SEM image (yarn cross section), and (v) Si elemental mapping (yarn cross section). (b) Screen-printed sample shown through (i) a photograph, (ii) a schematic, (iii) EDX surface analysis, (iv) SEM image (fabric cross section), and (v) Si elemental mapping (fabric cross section). (c) Dip-coated sample shown through (i) a photograph, (ii) a schematic, (iii) EDX surface analysis, (iv) SEM image (fabric cross section), and (v) Si elemental mapping (fabric cross section). (d) PU-bonded sample shown through (i) a photograph, (ii) a schematic, (iii) EDX surface analysis, and (iv) an SEM image (fabric cross section).

Surface topography and morphology of the samples were analyzed using scanning electron microscopy (SEM), while the presence of the triboelectric coating was confirmed by using the energy dispersive X-ray analysis (EDX) method (Figure 2a–d).

### Electrical Characterization

**Figure 3:**
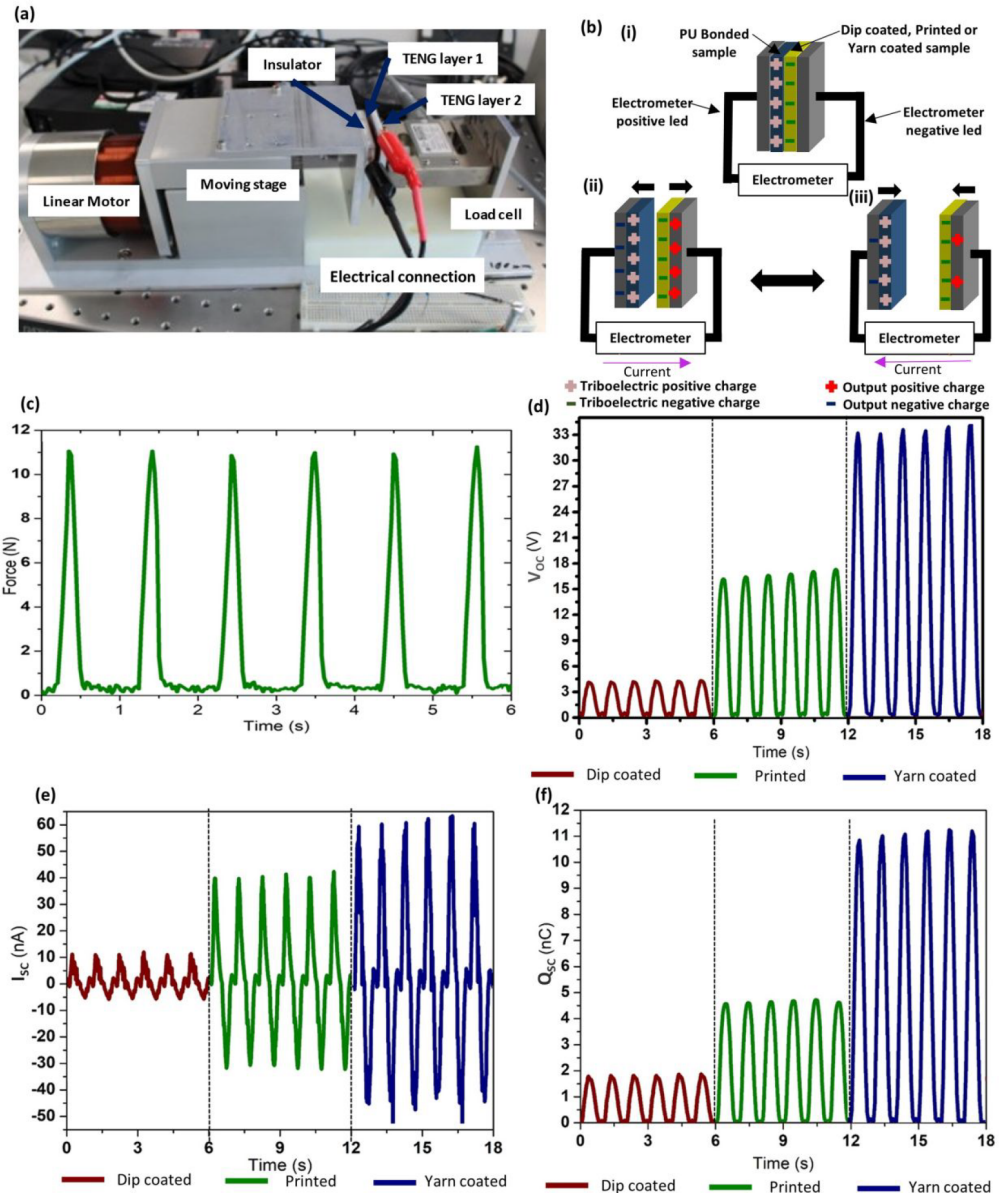
Electrical characterization of the TENGs. (a) A photograph of the TENG characterization setup. (b) Schematic of the working principle of the TENGs showing (i) the initial triboelectric charging, (ii) the separation half cycle, and (iii) the contact half cycle of TENG surfaces. (c) Contact force measurements during TENG layer movement cycles. (d,e) Electrical outputs (experimental) depicting the (d) *V*_oc_, (e) *I*_sc_, and (f) *Q*_sc_ values for the dip-coated, screen-printed, and yarn-coated TENG structures.

The electrical properties of the TENG were evaluated using a method similar to that reported in our previous work.^35−37^ A contact area of 5 cm × 5 cm was used for the electrical characterization. A bespoke linear motion system (Figure 3a,b) was used to provide contact and separation movements of triboelectric layer 1 and triboelectric layer 2 (1 mm amplitude, 1 Hz frequency, 10 N maximum contact force, and sinusoidal movement (Figure 3c)), and this process was followed to evaluate the outputs of the three application methods. Herein, we note that the selection of the maximum contact force (10 N) was based on our previous studies^2,25,35−37^ as well as contact force values used by other research groups^42−44^ for wearable TENG characterizations, representing an easily obtainable force/pressure value during regular movements for wearable TENG applications. In doing so, we hypothesize that these motion conditions closely represent the motion parameters that the textile TENG would be subjected to, in practical operating conditions.^9,10^ The outputs of the TENG surfaces (open circuit voltage (*V*_oc_), short circuit current (*I*_sc_), and short circuit charge (*Q*_sc_)) were measured using a Keithley 6514 electrometer.^2^ Furthermore, for the *V*_oc_, *I*_sc_, and *Q*_sc_ outputs, peak values of 20 output cycles were averaged. As discussed in our previous studies, the *V*_oc_, *I*_sc_, and *Q*_sc_ outputs offer substantial understanding of the performance of different TENGs,^35−37^ when measured under the same experimental parameters. These outputs, therefore, provide a strong basis for electrical output comparison between the fabrication methods during the present study, without needing to elaborately assess their power generation characteristics.

Because the scope of this study is to fundamentally compare the performance of the different TENG layers under typical room conditions, the electrical characterizations were conducted under temperature of 20 ± 2 °C, and relative humidity (RH) of 65 ± 4%, which are standard test conditions for textiles.

### Wearability Testing

Wearability of TENG layer 1 and TENG layer 2 was assessed by evaluating three key textile parameters, air permeability, moisture management, and stretch and recovery, subjected to standard textile testing conditions (24 h of preconditioning, temperature of 20 ± 2 °C, and relative humidity (RH) of 65 ± 4%). These wearability tests were conducted once the electrical characterizations have been completed to ensure the consistency and repeatability of the electrical outputs, because different wearability tests conducted in this work would require different samples sizes, stress, and moisture conditions and may involve changes to structural parameters.

Air permeability was measured using the British Standard BS 5636 with an SDL Atlas M021A air permeability tester (5 cm^2^ test area at 98 Pa). Moisture management properties were tested using an SDL Atlas moisture management tester, according to the American Association of Textile Chemists and Colorists Standard AATCC 195.^45^

The stretch and recovery test was conducted using an Instron tensile testing machine (4 cm × 8 cm fabric area connected to a 2-in. gauge length and subjected to a maximum load of 34 N) from which the stretchability, maximum stretch, elastic modulus, and recoverability were recorded.

## Results and Discussion

The surface topography and morphology of the yarn-coated (Figure 2a,i–v), screen-printed (Figure 2b,i–v), dip-coated (Figure 2c, i–v), and PU-bonded (Figure 2d,i–iv) TENG layers were observed using SEM and EDX techniques. The SEM (Figure 2a,iv) and EDX (Figure 2a,v) analyses of the cross section of the yarn used for the yarn-coated TENG demonstrate that the conductive silver yarn is fully covered with PDMS, creating a core–shell structure. This full encapsulation of the conductive yarn with PDMS is considered critical in reducing the chance of possible charge leakages during triboelectric contact with the TENG layer 2. For the scope of the present experimental work, this PDMS coating, which fully covers the conductive surface of the yarns, is considered sufficient; however, we note that the evenness and the uniformity of such coatings or coverings can further be improved using advanced yarn processing techniques such as the yarn sizing process, bicomponent spinning, modified rotor spinning, and hollow spindle covering.^46−49^

As depicted in the SEM image (Figure 2a,iv), the PDMS coating around the yarn has an average thickness of ∼309.6 μm. As per the EDX analysis (Figure 2a,iii), this contains a Si weight percentage of 69%. The SEM and EDX images of the screen-printed TENG layer (Figure 2b,iv,v) show that the PDMS coating is limited to the top surface of the fabric, while the EDX analysis (Figure 2b,iii) indicates a Si percentage of 19.8%. Furthermore, the SEM (Figure 2c,iv) and EDX (Figure 2c,v) outcomes of the dip-coated TENG layer exhibit complete encapsulation of the fabric with PDMS from the top and bottom surfaces, including infiltration of the PDMS into the fibers, while the EDX report indicates a Si percentage of 26%. On the other hand, analysis of the secondary triboelectric surface demonstrates that the thickness of PU bonding on the average is around 20.9 μm, and the PU material is concentrated on the topmost layers of the conductive fabric. Therefore, the SEM and EDX analyses confirmed that these TENG surfaces comply with the industry related norms, where (i) the coating material is encompassing the yarn during yarn coating, (ii) the coating material is restricted to the fabric front surface during screen printing and boding, and (iii) the coating material encapsulates the fabric from both the top and the bottom surfaces (face and back side) during dip coating, respectively.

Furthermore, with regards to the thickness variations of each coating method, we would note the following. The objective of this study was to use yarn-coating, screen-printing, and dip-coating techniques equivalent to the industrial fabrication methods, and to observe the output trends of the fabricated TENG layers. The thickness variations observed during the three techniques are inherent to the coating methods themselves, as yarn coating typically leads to more uniform and lower coated thickness, followed by screen printing and dip coating that provide thicker and coarser coated thicknesses. Hence, we note that the variation in the thickness of triboelectric coatings is inherent to the coating methods, which could affect the electrical performances of the respective TENG layers.

### Electrical Characterization

As explained in the Experimental Methods, TENG layer 1 and TENG layer 2 were subjected to vertical contact and separation movements for each of the coating methods, and the corresponding outcomes are depicted in Figure 3d–f. Looking at their position in the triboelectric series, PU is ranked higher in comparison to PDMS. Therefore, upon contacting with each other, the PU surface gets triboelectrically charged positively, whereas the PDMS surface gets triboelectrically charged negatively. In the electrical measurement system used in this work, the positive lead of the electrometer is connected to the electrode (conductive fabric) of the PU TENG surface, and the negative lead is connected to the electrode (conductive fabric/yarns) of the PDMS TENG surface. Initially, when the two triboelectric surfaces are in contact, both positive and negative triboelectric charges are on the same plane; therefore, there is no output from the TENG device (Figure 3b,i). However, when the triboelectric surfaces are separated, the positively charged PU surface induces a positive potential on its electrode, whereas the PDMS surface induces a negative potential on its electrode (Figure 3b,ii). This creates a charge flow between these electrodes, resulting in a positive increase in charge (Figure 3f) and voltage (Figure 3d), as well as a positive current pulse (Figure 3e). When the surfaces are moved toward contact, this process reverses, creating a charge flow in the opposite direction (Figure 3b,iii). Therefore, both the charge (Figure 3f) and the voltage outputs (Figure 3d) return toward zero, and a negative current pulse is recorded (Figure 3e). Upon continuously contacting and separating the triboelectric surfaces, an alternating electrical output can be obtained. Depending on the TENG layer type, different electrical outputs are observed, as seen from Figure 3d–f, which can be analyzed as follows.

Considering *V*_oc_, the yarn-coated TENG layer demonstrates the best performance with a peak voltage of around 34.5 V (Figure 3d). The peak *V*_oc_ values for the screen-printed TENG layer and the dip-coated TENG layer were approximately 17.3 and 4.9 V, respectively. Furthermore, the yarn-coated TENG layer showed a peak *I*_sc_ of ∼60 nA, the screen-printed TENG showed ∼43 nA, and the dip-coated TENG showed ∼11 nA (Figure 3e). A similar trend was observed for the *Q*_sc_ outcomes, where the highest charge output was recorded for the yarn-coated TENG (∼12 nC), followed by the screen-printed TENG (∼5 nC) and the dip-coated TENG (∼2 nC) (Figure 3f).

**Figure 4:**
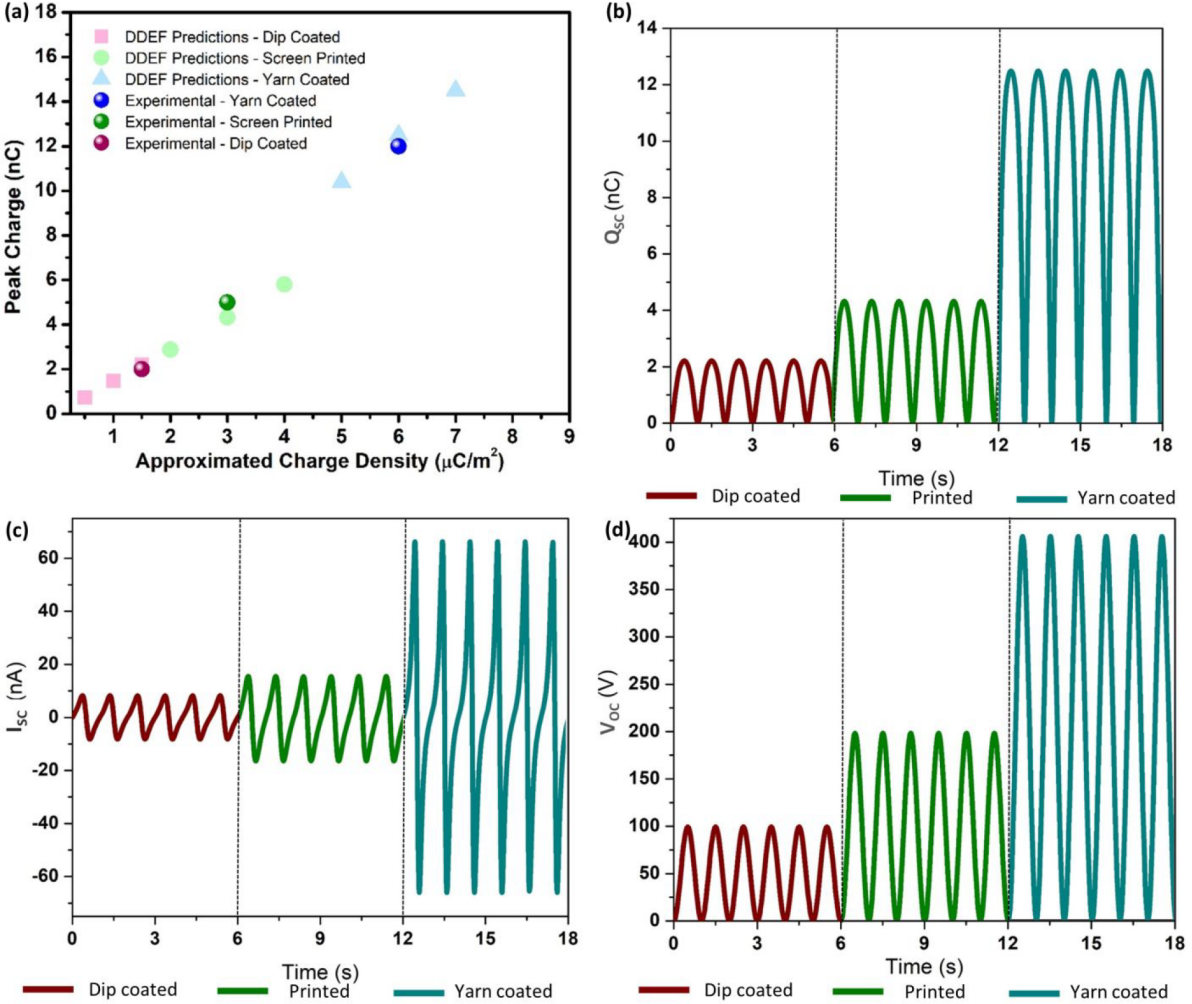
DDEF model simulations for the electrical outputs of the TENG structures. (a) Approximation of the theoretical charge density for each TENG based on the experimental peak charge. Output simulations for (b) *Q*_sc_, (c) *I*_sc_, and (d) *V*_oc_ for the yarn-coated, screen-printed, and dip-coated TENG structures.

Following the experimental characterization, the distance-dependent electric field (DDEF) model was used to simulate the output trends of the TENGs (Figure 4). A description of the techniques and the parameters used for these simulations is provided in the Supporting Information section S1. As the first step, the theoretical triboelectric charge density, which matches the experimental peak *Q*_sc_ for each TENG type, was evaluated, indicating an approximated charge density of 1.5 μC/m^2^ for the dip-coated TENG, 3 μC/m^2^ for the screen-printed TENG, and 6 μC/m^2^ for the yarn-coated TENG (Figure 4a). The corresponding DDEF model simulations show a trend similar to that of the experimental outputs for the *Q*_sc_ (Figure 4b), *I*_sc_ (Figure 4c), and *V*_oc_ outputs.

We note that the difference between the amplitude of the experimental and simulated voltages is observed throughout all theoretical models to date^35−37,50,51^ and is believed to have been caused by the excessive internal impedance of the TENG during the experimental measurements.

The reasons behind the experimental and theoretical output patterns for the yarn-coated, screen-printed, and dip-coated TENG surfaces observed during the electrical characterization can be hypothesized as follows. Among the primary parameters affecting the triboelectric outputs, the contact surface area holds a key importance as it directly impacts the triboelectric charging process.^35−37^ This is in turn affected by the nature of the contact surfaces and the uniformity of the triboelectric coatings on each surface. Furthermore, the distance between the triboelectrically charged surface and the electrode has a significant impact on the output induction, where thinner coatings tend to have improved output induction, and hence better output performances.^36^

The higher output generation of the yarn-coated TENG surface can be explained on the basis of two major parameters. First, this is affected by the relatively low thickness of the dielectric component (PDMS) of the TENG layer. The PDMS coating, as previously stated, is around ∼309 μm; therefore, the distance between the triboelectric charges and the electrode (in this case, the silver-coated surface of the yarn) is minimum. This would facilitate maximum electric field propagation to the electrode–dielectric interface, and thus maximum output induction among the three techniques used in the study. Second, a rib fabric structure (fabric used in this study) is constructed using double needle-bed knitting, where alternating sets of loops are pulled toward the front and back needle beds. As the fabric relaxes, the set of loops (technically termed as courses in knitting), which were constructed by the front needle bed, tends to appear on one side of the fabric, whereas the back needle bed loops appear on the opposite side. When the compression force (∼10 N) is applied on this fabric during the contact-separation movement of the TENG, the yarn-coated TENG layer would expand due to its structure and loop mobility, potentially bringing both the front and the back courses on to contact with the PU surface. This would facilitate improved contact between TENG layer 1 and TENG layer 2. The DDEF model (Supporting Information section S1) simulation supports this hypothesis, suggesting the highest theoretical charge density for the yarn-coated TENG structure.

However, the thicknesses of the dip-coated and (1.35 mm) screen-printed (1.27 mm) TENG surfaces are significantly higher than that of the yarn-coated scenario (0.3 mm), which would reduce their output generation as compared to the yarn-coated TENG.

Comparing the screen-printed and the dip-coated TENG surfaces, first, the screen-printed TENG layer has a relatively lower thickness, which thus would result in a higher output induction as compared to the dip-coated layer. Additionally, the screen-printed TENG layer contains a relatively uniform and even contact surface, whereas the dip-coated TENG layer is uneven due to a low degree of control and nonuniformity inherent to the dip-coating process, especially with regards to viscous liquids such as PDMS. This would negatively affect the triboelectric contact as well as the charging behavior of the dip-coated TENG surface. Consequently, the screen-printed TENG structure produces higher outputs than does the dip-coated TENG structure (Figures 3 and 4).

From the above analysis, it is evident that the yarn-coated TENG layer provides the best electrical output performance out of the fabrication methods used in this study. It should be noted that, for the scope of this Article, fabrication of PU-coated TENG (TENG layer 2) was kept consistent, to obtain a systematic comparison of electrical outputs for PDMS-coated TENG layers fabricated using different techniques. It could further be hypothesized from the above results that, if the PU was applied on TENG layer 2 via yarn coating process, it could result in more improved electrical outputs.

### Wearable Characterization

**Figure 5:**
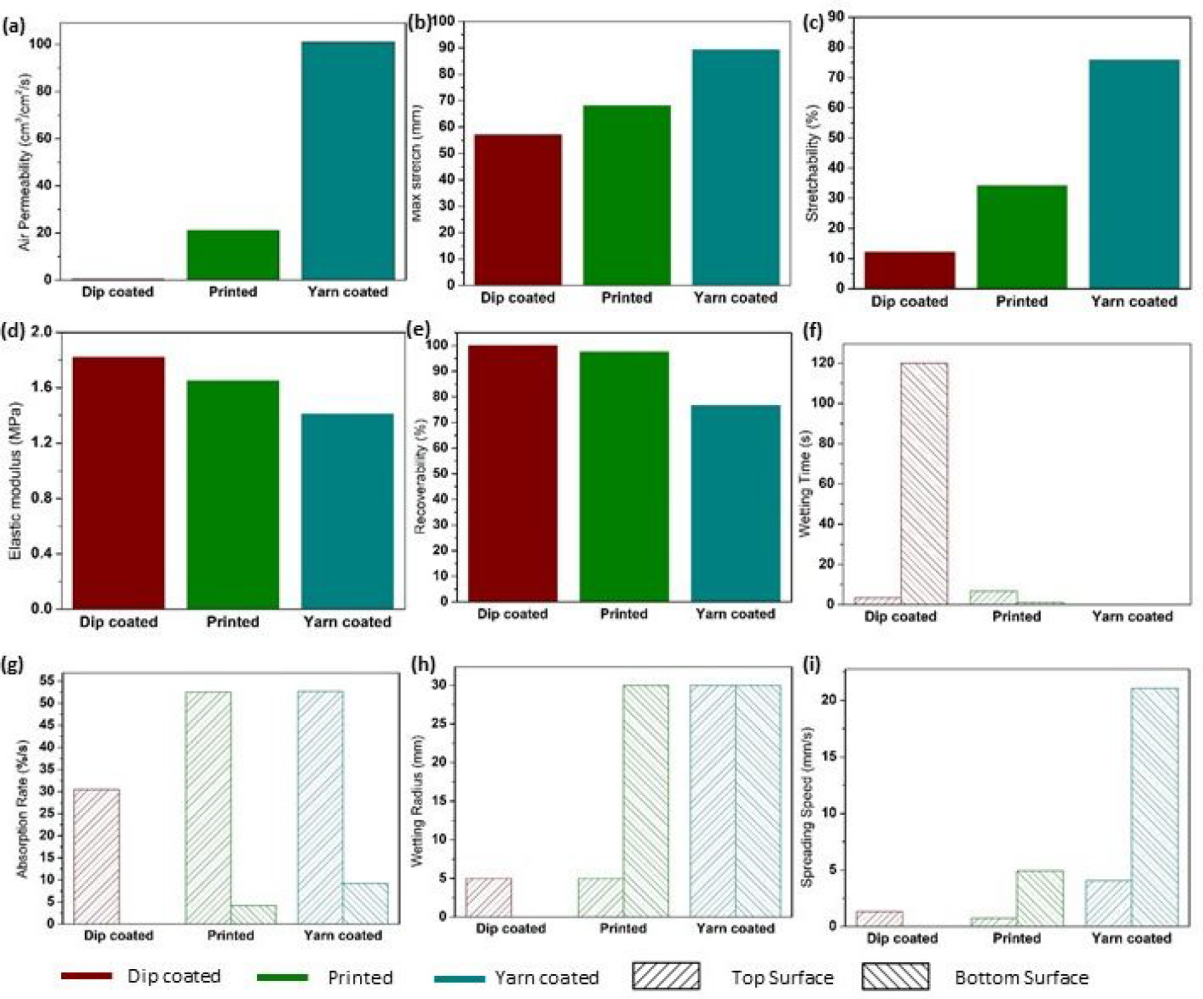
Characterization of the wearable properties of the TENG surfaces, indicating (a) air permeability, (b) maximum stretch, (c) stretchability, (d) elastic modulus, (e) recoverability, (f) wetting time, (g) absorption rate, (h) wetting radius, and (i) spreading speed.

Several key textile properties that affect the wearability of textile TENG devices were investigated following the electrical characterization. Air permeability, which measures the flow of air through a given fabric area, relates directly to the thermal comfort of a textile. High air permeability of a textile (as an example, for workout and outwear garments) leads to quick drying of the fabric, providing a high degree of comfort to the wearer.^52^ Subjected to the standard air permeability test (BS 5636 (currently revised as ISO 9237^53^)), the yarn-coated TENG layer demonstrated a superior air permeability value of 101 cm^3^/cm^2^/s, whereas the screen-printed and dip-coated TENG layers provided relatively low permeability values of 21.2 and 0.551 cm^3^/cm^2^/s, respectively (Figure 5a). As is evident from the optical and SEM analysis (Figure 2a), the yarn-coated fabric contains openings in its knitted yarn structure resulting in high yarn porosity, which causes high air permeability. The screen-printed TENG surface contains a denser surface coating, which blocks many of the openings of the fabric from the top surface, and therefore shows a moderate air permeability. The dip-coated sample has a thick coating completely encompassing the fabric, which results in a very low air permeability.

Stretchable fabrics can extend under applied force, and any wearable textile should contain an adequate degree of stretch to facilitate easy and comfortable body movements. To evaluate the stretch of the fabric, the maximum total stretch (Figure 5b), the stretchability (defined as (maximum stretch – initial length) × 100/initial length) (Figure 5c), and the elastic modulus (Figure 5d) of 50 mm TENG fabric samples were measured. The highest stretchability among the TENG surfaces was recorded for the yarn-coated TENG surface, with a stretchability of ∼75% (maximum stretch up to 89.32 mm) (Figure 5b,c). Comparatively, the screen-printed sample showed a stretchability of 34.26% (maximum stretch up to 68.21 mm), while the stretchability of the dip-coated sample was 12.18% (maximum stretch up to 56.99 mm) (Figure 5b,c). An opposite trend can be observed in the elastic modulus of the TENG surfaces, with the yarn-coated TENG reporting 1.4 MPa, screen-printed TENG giving 1.7 MPa, and the dip-coated TENG reporting 1.8 MPa (Figure 5d). In other words, this means that the yarn-coated TENG layer deforms more under an applied load (low modulus), whereas the dip-coated TENG layer shows the minimum deformation (high modulus). The rib knit structure used in this work is an inherently stretchable fabric, and the yarn-coating technique helps to retain these excellent stretch properties due to the mobility between the knitted loops, which results in the high stretchability, maximum stretch, and low elastic modulus. However, in the case of screen-printed TENG, this mobility is restricted due to the top surface coating of the PDMS, bringing its stretchability and modulus down to a moderate level. Furthermore, the relatively high overall PDMS coating on the dip-coated sample on both of its surfaces restricts the movement of the dip-coated fabric, resulting in the lowest stretchability and highest elastic modulus.

Furthermore, recoverability is an important property of textiles where the ability to recover from applied pressure/stretch is measured to assess their capability to retain original dimensions and shape. The dip-coated TENG layer, due to its relatively high rigidity, shows the highest recovery (100%) after being subjected to the maximum stretch, followed by the screen-printed (97.5%) and yarn-coated (76.67%) TENG layers (Figure 5e). While the recovery of the yarn-coated TENG textile at very high extensions is comparatively low, it can be still be categorized as a moderate and sufficient recovery behavior for typical textile applications as well as for smart textile applications. Furthermore, this recovery behavior can further be improved by controlling the thickness of the triboelectric coating and through structural modifications to the knitted fabric.^54,55^

Moisture management of a textile assesses its ability to absorb and draw moisture away from the skin of the wearer, which critically affects the comfort properties. This is measured through parameters such as the wetting time (Figure 5f), the absorption rate (Figure 5g), the wetting radius (Figure 5h), and the spreading speed (Figure 5i). During standard testing, water droplets are put on the fabric top surface (in this case, the triboelectric contact surface of the TENG), and the aforementioned parameters are assessed for the fabric top surface (triboelectric contact surface of the TENG layer) and the bottom surface (noncontact surface of the TENG layer). In this work, PDMS, which is typically a hydrophobic coating (similar to many other common textile coatings), was used as the triboelectric coating material, and it was observed that the coating method has a significant influence on its moisture management characteristics.

The wetting time test (Figure 5f) assesses the time taken for the contact angle between a water droplet and a fabric surface to reach a threshold (tangential of more than 15°), providing an idea about the ease of fabric wetting and how fast it absorbs moisture. Considering the dip-coated TENG surface, both the top (contact side of the TENG layer) and the bottom surfaces (noncontact side of the TENG layer) did not demonstrate notable wetting, because both sides of the fabric were covered with PDMS, thus effectively blocking the channels (pores) in the fabric structure, which would absorb and transport water. However, the screen-printed TENG surface demonstrated a relatively better wetting time due to its relatively thinner PDMS coating as compared to the dip-coated TENG layer, and the relatively higher structural openings, which allowed channels for the moisture to be transported. The top surface of the screen-printed TENG showed a moderate wetting time, whereas the bottom surface (opposite to the printed side) showed a low wetting time. The yarn-coated TENG layer demonstrated the best performance for the wetting time due to its high structural openness, wicking properties, and low thickness, allowing the moisture to be transported easily on both sides of the fabric.

The absorption rate (amount of water absorbed by fabric as a percentage to the amount of water dropped to the system) test measures the average rate of moisture absorption of a textile surface (Figure 5g). Because of reasons similar to those in the case of wetting time, the dip-coated TENG layer shows a low absorption rate. In comparison, the screen-printed TENG surface shows a relatively improved absorption rate, while the TENG surface demonstrates the best absorption rate.

The maximum wetting radius and the spreading speed of a liquid on a fabric surface depend on its capillary action and wicking properties, which are important to draw moisture away from the skin during wearable applications. Considering the performance of both the top and the bottom fabric surfaces, the dip-coated TENG demonstrates the weakest performance in both the wetting radius and the spreading speed (Figure 5h,i). The screen-printed TENG surface shows a moderate overall performance, whereas the best performance is seen from the yarn-coated TENG surface.

The same wearability tests were conducted for the TENG layer 2 (PU-bonded TENG surface), and a relevant analysis is presented in section S2 of the Supporting Information.

Overall, the yarn-coated TENG surface demonstrates a balanced performance, showing improved electrical outputs (*V*_oc_, *I*_sc_, and *Q*_sc_) as well as improved wearable characteristics including the air permeability, moisture management, and stretch properties. The durability of the electrical properties is also visible throughout a considerable number of process cycles (Supporting Information section S3). The outcome of this work demonstrates that, similar to the material selection and the fabric structure selection, the application method of the triboelectric materials into the textiles plays a key part in their output performances. Furthermore, as predicted in our previous work,^1^ functionalizing the textile building blocks, i.e. textile fibers/yarns, is an efficient method not only to improve their electrical outputs, but also to significantly improve their wearable characteristics, which are essential for future wearable applications.

## Conclusions

This work evaluates the effectiveness of some the most common textile manufacturing techniques, yarn coating, dip coating, and screen printing, as scalable fabrication methods for wearable TENGs. Textile TENG layers, constructed using typical textile materials and acting as triboelectric surfaces, were assessed for their electrical performances as well as their wearable characteristics using standard test procedures. In terms of electrical outputs, the yarn-coated TENG architecture provides a maximum performance of *V*_oc_ ≈ 35 V, *I*_sc_ ≈ 60 nA, and *Q*_sc_ ≈ 12 nC (for a 5 cm × 5 cm surface area) when subjected to a 1 mm amplitude and 1 Hz frequency sinusoidal contact-separation movement. In comparison, the screen-printed TENG layer provided a performance of *V*_oc_ ≈ 17 V, *I*_sc_ ≈ 43 nA, and *Q*_sc_ ≈ 5 nC, whereas the dip-coated TENG layer demonstrated *V*_oc_ ≈ 5 V, *I*_sc_ ≈ 11 nA, and *Q*_sc_ ≈ 2 nC. Similarly, the yarn-coated TENG layer demonstrated significantly better wearable performances in terms of moisture management, air permeability, and stretch properties, implying a higher degree of comfort for wearable applications. Therefore, the outcomes of this study provide a platform and guidelines toward the future design and fabrication of efficient wearable TENG architectures, which contain a balance of both electrical and wearable performances.

## Supporting Information Available

Section S1, parameters for the DDEF model simulations of textile TENGs; section S2, wearability characterization of the PU TENG layer (triboelectric layer 2); and section S3, endurance analysis for the yarn-coated sample (PDF)
