# Supplementary material for: Scalable Textile Manufacturing Methods for Fabricating Triboelectric Nanogenerators with Balanced Electrical and Wearable Properties
Source: ACS Appl Electron Mater. 2022 Jan 26;4(2):678–88. doi: 10.1021/acsaelm.1c01095 (PMC9097478; doi:10.1021/acsaelm.1c01095)
Supplement: Supplementary file 1 — el1c01095_si_001.pdf [file el1c01095_si_001.pdf]

Supporting Information

Scalable Textile Manufacturing Methods for Fabricating Triboelectric Nanogenerators with Balanced Electrical and Wearable Properties

*K. R. Sanjaya Gunawardhana [a], Nandula D. Wanasekara [a *], Kahagala Gamage Wijayantha [b], R. D. Ishara Dharmasena [a, c *]*

a - Department of Textile and Apparel Engineering, Faculty of Engineering, University of Moratuwa, Bandaranayake Mawatha, Moratuwa, 10400, Sri Lanka

b - Energy Research Laboratory, Department of Chemistry, Loughborough University,

Loughborough, Leicestershire, LE11 3TU, United Kingdom.

c - Wolfson School of Mechanical Electrical and Manufacturing Engineering, Loughborough University, Loughborough, Leicestershire, LE11 3TU, United Kingdom.

Corresponding Authors: R. D. Ishara Dharmasena (r.i.dharmasena@lboro.ac.uk)

Nandula D. Wanasekara (nandulad@uom.lk)

S1

The theoretical simulations of the TENG surfaces were conducted using the distance-dependent electric field (DDEF) theoretical platform.[1–4] The distance-dependent electric field equation (eqn. S_1) considers a triboelectrically charged surface with length L, width W, surface charge density σ, which is placed in a medium with permittivity ε, and approximates the average electric field of this surface above its midpoint along an axis perpendicular to the surface (E_x), as follows:

$$E_x = \frac{\sigma}{\pi\varepsilon}\arctan\left(\frac{L/W}{2\left(x/W\right)\sqrt{4\left(x/W\right)^2 + \left(L/W\right)^2 + 1}}\right) = \frac{\sigma}{\pi\varepsilon}f(x) \qquad \text{(S\_1)}$$

where x is the perpendicular distance in consideration from the charged surface.

By applying eqn. S1 to the triboelectric contact surfaces and the electrodes of the textile TENG structures, the DDEF model for the textile TENG surfaces was derived, which was then used to approximate the outputs of the TENG architectures.

Some of the key parameters used for the different TENG surfaces are summarised below:

L = W = 50 mm

Dielectric constant of PDMS = 2.7

Dielectric constant of PU = 3.9

Thickness of the yarn coated surface = 0.3 mm

Thickness of the screen-printed surface = 1.27 mm

Thickness of the dip coated surface = 1.35 mm

Thickness of the PU bonded surface = 0.1 mm

S2

Wearability performance analysis for PU bonded triboelectric layer (TENG layer 2). All the tests shown here were conducted using the same test standards and methods followed during the testing of TENG Layer 1.

Property	Subcategory	Value	
Air permeability		0.904 $cm^3/cm^2/s$	
Stretch and Recovery	Max Stretch	80.725 mm	
	Stretchability	58.9 %	
	Recoverability	69.28%	
	Elastic modulus	4.5123 MPa	
Moisture Management properties		**Top Surface**	**Bottom Surface**
	Wetting time	1.219 s	11.344 s
	Absorption rate	30.1472 % s^{-1}	7.4177 % s^{-1}
	Max wetting radius	25 cm	15 cm
	Spreading speed	4.7545 mm/s	2.0983 mm/s

Table: Wearability performance analysis for the PU bonded triboelectric layer

Considering the outputs of the wearability tests, the PU bonded TENG layer shows low air permeability, moderate stretch and recovery as well as moisture management properties.

S3

Durability analysis for yarn coated sample, showing the Open circuit voltage outputs in day 1 (initial testing) and in day 2 (after 3000 contact separation cycles).

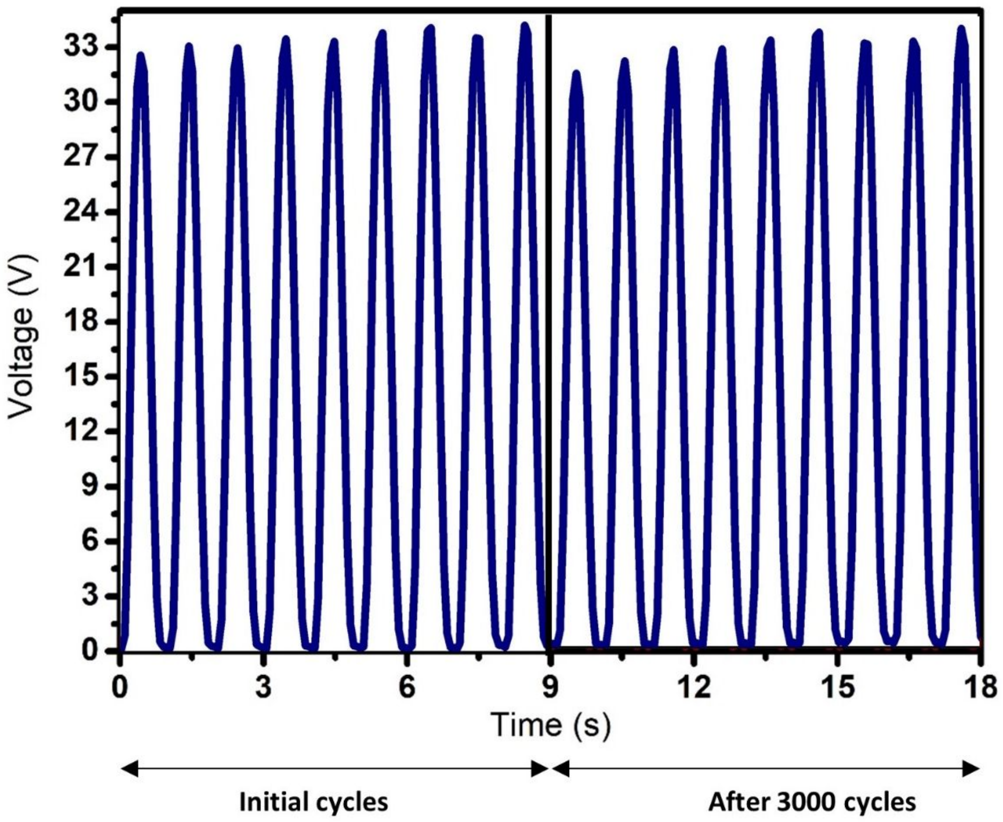

Figure: Durability analysis of the yarn coated sample

References:

(1) Dharmasena, R. D. I. G.; Jayawardena, K. D. G. I.; Mills, C. A.; Deane, J. H. B.; Anguita, J. V.; Dorey, R. A.; Silva, S. R. P. Triboelectric Nanogenerators: Providing a Fundamental Framework. *Energy Environ. Sci.* **2017**, *10* (8), 1801–1811. https://doi.org/10.1039/C7EE01139C.

(2) Dharmasena, R. D. I. G.; Jayawardena, K. D. G. I.; Mills, C. A.; Dorey, R. A.; Silva, S. R. P. A Unified Theoretical Model for Triboelectric Nanogenerators. *Nano Energy* **2018**, *48*, 391–400. https://doi.org/10.1016/j.nanoen.2018.03.073.

(3) Dharmasena, R. D. I. G.; Deane, J. H. B.; Silva, S. R. P. Nature of Power Generation and Output Optimization Criteria for Triboelectric Nanogenerators. *Advanced Energy Materials* **2018**, 1802190. https://doi.org/10.1002/aenm.201802190.

(4) Dharmasena, R. Inherent Asymmetry of the Current Output in a Triboelectric Nanogenerator. *Nano Energy* **2020**, 105045.
